# Preliminary Kinetic Assessment of Quality Deterioration and Shelf-Life Estimation in Yellowfin Tuna Under Different Constant Storage Temperatures

**DOI:** 10.3390/foods15162829

**Published:** 2026-08-14

**Authors:** Jianchao Deng, Tianyu Chen, Jin Huang, Chunsheng Li, Xiao Hu, Xue Zhao, Yanyan Wu, Shengjun Chen, Yongqiang Zhao

**Affiliations:** 1 Key Laboratory of Aquatic Product Processing, Ministry of Agriculture and Rural Affairs, National R&D Center for Aquatic Product Processing, South China Sea Fisheries Research Institute, Chinese Academy of Fishery Sciences, Guangzhou 510300, China; djc9801@foxmail.com (J.D.); chentianyu5220@stu.ouc.edu.cn (T.C.); hnhuxiao@163.com (X.H.); shirlizhao@163.com (X.Z.); wuyygd@163.com (Y.W.); chenshengjun@scsfri.ac.cn (S.C.); 2 Key Laboratory of Efficient Utilization and Processing of Marine Fishery Resources of Hainan Province, Sanya Tropical Fisheries Research Institute, Sanya 572018, China; 3 College of Food Science and Engineering, Ocean University of China, Qingdao 266003, China; 4 College of Food Science & Technology, Shanghai Ocean University, Shanghai 201306, China; 13939791325@163.com

**Keywords:** yellowfin tuna, biogenic amine, total volatile basic nitrogen, total plate count, shelf life

## Abstract

The quality of yellowfin tuna is highly vulnerable to microbial proliferation, which accelerates quality deterioration and spoilage throughout storage. In this study, the dynamic changes in biogenic amines (BAs), total volatile basic nitrogen (TVB-N), and total plate count (TPC) across a different storage temperature range of 0 to 25 °C were studied, followed by a shelf-life prediction based on kinetic models. The TPC, TVB-N, and most BAs increased significantly with increasing storage time and temperature. Correlation analysis was used as an exploratory approach and showed that only histamine and cadaverine exhibited significantly positive correlations (|r| ≥ 0.6, *p* < 0.05) with TPC and TVB-N within the present dataset. Histamine was selected as a candidate BA indicator for kinetic modeling because only this BA changed significantly during storage at different temperatures. Utilizing histamine, TPC, and TVB-N, preliminary kinetic equations for shelf-life estimation were developed using first-order kinetics and the Arrhenius equation. Among the tested indicators, TPC showed relatively greater temperature sensitivity and preliminary within-batch agreement. These results provide preliminary reference data for further validation of tuna during the refrigeration process.

## 1. Introduction

Yellowfin tuna (*Thunnus albacares*) is a high-value seafood widely traded in global markets [1]. Yellowfin tuna is abundant in proteins, free amino acids, and nitrogenous substances, which readily facilitate microbial propagation and endogenous enzymatic reactions, and eventually trigger spoilage. The deterioration of tuna quality during distribution and storage is predominantly driven by microbial growth and the occurrence of biochemical reactions [2]. This deterioration not only leads to quality loss but also increases the risk of foodborne illness [3]. Therefore, yellowfin tuna is usually preserved under frozen conditions during transportation and storage to prevent quality deterioration and extend shelf life [4]. Nevertheless, unavoidable temperature fluctuations along the yellowfin tuna supply chain substantially shorten its shelf life. For example, yellowfin tuna is commonly consumed as sashimi, and significant temperature fluctuations occur during this process. Therefore, the industry requires quantifiable and predictable shelf-life tools for quality control and temperature control decisions, especially for shelf-life prediction at temperatures above the freezing point.

Fish spoilage is a multi-factor process driven primarily by microbial growth and the associated biochemical transformations of proteins and lipids [5]. The common indicators for shelf-life prediction of aquatic products include pH, total plate count (TPC), basic nitrogen (TVB-N), and TBARs [6,7]. TPC is commonly used to characterize the overall microbial proliferation that underlies spoilage progression. As early as 2005, Guizani et al. explored the changes in histamine and TPC in yellowfin tuna at different storage temperatures [8]. TVB-N, comprising volatile basic compounds such as ammonia and amines generated through protein degradation and microbial metabolism, is widely applied as a chemical freshness index for marine products [9]. Yellowfin tuna, as an amine-producing fish, produces a large amount of biogenic amines (BAs) when stored at temperatures above the freezing point. Therefore, BAs are other important predictors for amine-producing fish. Bas, including histamine, putrescine, spermine, tyramine, cadaverine, and spermidine, are produced by the decarboxylation of free amino acids in fish by amino acid decarboxylase in microorganisms [10] and cause a series of poisoning symptoms after excessive intake by humans [11]. Histamine, possessing the most abundant and intense toxicity in tuna, is one of the primary obstacles to export trade [8]. Therefore, histamine is one of the important indicators for shelf-life prediction because it is directly related to the freshness of fish [12].

Although these indicators are widely used for predicting the shelf life of yellowfin tuna, there are still many challenges in applying them to build an expiration date model [13]. Under different processing conditions and storage temperatures for tuna, the sensitivity and reliability of individual indicators may vary [14]. Moreover, these indicators are not independent of each other. The growth of microorganisms simultaneously accelerates the accumulation of TVB-N and the formation of BAs [15]. Improper selection of freshness indicators can result in overcomplicated or low-sensitivity prediction models [16]. Therefore, it is necessary to conduct indicator screening before determining the shelf life of yellowfin tuna under different temperature conditions.

Dynamic modeling provides an effective framework that can convert the changes in quality into quantitative shelf-life predictions [16]. To describe the loss of freshness and the change in shelf life of seafood during storage, classical kinetic methods are often adopted, including zero-order and first-order equations combined with terms describing temperature dependence based on the Arrhenius equation. These methods have been widely used to simulate the changes in microbial counts, TVB-N, K value, lipid oxidation indexes, and other quality characteristics of aquatic products [16,17]. Zero-order kinetics assume that the deterioration rate is independent of the current quality level, while first-order kinetics assume that the rate is proportional to the current concentration or the remaining deterioration potential. Both of these methods simplify the complex microbial, enzymatic and biochemical spoilage processes into apparent rate constants. Similarly, the Arrhenius model is applicable to controlling temperature-dependent reaction rates under isothermal conditions [18]. The classical kinetic models have few parameters and strong interpretability, making them suitable for initially describing the temperature-dependent changes under specific experimental conditions.

In this study, the contents of BAs, TVB-N and TPC during storage were determined, and the relationships among them were analyzed using relevant heat maps and network analysis to identify the most important indicators during the deterioration of yellowfin tuna quality. On this basis, first-order kinetic models were constructed to quantify variations in screened key BAs, TVB-N and TPC at five storage temperatures, followed by validation tests to assess model performance and practical applicability.

## 2. Materials and Methods

### 2.1. Materials

Ultra-low-temperature frozen yellowfin tuna was purchased from a local aquatic product retailer in Guangzhou, China. All yellowfin tuna came from the same batch. The yellowfin tuna was immediately frozen at −70 °C after being caught and transported below −20 °C. After purchase, the tuna was placed in an insulated box and transported to the laboratory within 1 h. Standard amines including putrescine, cadaverine, spermine, spermidine, tryptamine, phenylethylamine, histamine, tyramine and dansyl chloride were purchased from Sigma-Aldrich (St. Louis, MO, USA). Perchloric acid, hydrochloric acid, acetone, sodium hydroxide, sodium bicarbonate, ammonia boric acid, anhydrous ethanol, phenolphthalein, methyl red, magnesium oxide, methylene blue and sodium chloride were acquired from Aladdin (Shanghai, China). Agar culture mediums were prepared by Huayu Biotechnology Ltd. (Guangzhou, China). Acetonitrile used in this experiment was HPLC-grade, and ultrapure water was prepared using a Millipore ultrapure water purification system (Bedford, MA, USA).

### 2.2. Preparation of Yellowfin Tuna Samples

After the yellowfin tuna was thawed, the back muscle was cut into slices (5 × 5 × 2 cm) and vacuum-packed. Then, the samples were stored at 0 °C, 4 °C, 10 °C, 20 °C and 25 °C, respectively. At 25 °C, samples were taken after 0.125, 0.25, 0.5, 1, 1.5, 1.75 and 2 d of storage. At 20 °C, samples were taken after 0.5, 1, 1.5, 2, 2.5, and 3 d of storage. At 10 °C, samples were taken after 1, 2, 3, 4, 5, 6 and 7 d of storage. At 4 °C, samples were taken after 1, 2, 3, 4, 5, 6 and 7 d of storage. At 0 °C, samples were taken after 2, 4, 6, 8, 10, 12 and 14 d of storage.

### 2.3. Determination of TVB-N

The determination method of TVB-N was based on a previous study with minor modifications [5]. Briefly, a 20 g minced tuna sample was homogenized with 100 mL of distilled water and soaked for 30 min prior to filtration. A mixture of 10 mL sample filtrate and 5 mL of MgO suspension was distilled. Subsequently, the titration solution was used to determine the titration endpoint, and the consumed volume of the titration solution was recorded.

### 2.4. Determination of TPC

The determination method of TPC was based on a previous study with minor modifications [19]. In order to transfer the microorganisms from the yellowfin tuna to the liquid, the minced tuna was homogenized with sterile normal saline at a 1:10 ratio, and 1 mL of the homogenate was subjected to serial gradient dilutions. Two appropriate dilutions were incubated at 30 °C for 72 h, and the total plate count was enumerated afterwards.

### 2.5. Determination of BAs

The detection of BAs in tuna was carried out according to the method proposed by Zhao et al. [20]. The 4.00 g sample was mixed with 10 mL of 0.4 mol/L perchloric acid, and then the homogenate was centrifuged at 5000 rpm for 10 min. The supernatant was collected in a 25 mL brown volumetric flask. The residue was re-extracted with perchloric acid, and then the mixture was centrifuged. The combined supernatants were diluted. For derivatization, NaOH and saturated sodium bicarbonate were sequentially added to a 1 mL aliquot, and then 2 mL of dansyl chloride (10 mg/mL) were added. Subsequently, an ammonia solution was used to terminate the reaction after derivatization in the darkness. The mixture was diluted to 5 mL with acetonitrile and filtered through a 0.22 μm filter membrane. The prepared filtrate was used for the HPLC analysis. Standard solutions were processed identically.

Separation was carried out using the WondaCract ODS-2 (250 mm × 4.6 mm × 5 μm) column. The injection volume was 10 μL, and the column temperature was 35 °C. The mobile phases A and B were 0.01 mol/L ammonium acetate and water + acetonitrile (1:9), respectively, and the flow rate was 1.0 mL/min. The gradient elution program was set as follows: 0–5 min, 55% B; 25–35 min, 55–95% B; 35–43 min, 95–55% B; 43–45 min, 55% B. The detection limit and quantification limit for BAs in aquatic products and meat were 20 mg/kg and 50 mg/kg respectively. The precision requirement was that the absolute difference between the two independent measurement results obtained under the same repeatability conditions should not exceed 10% of the arithmetic mean.

### 2.6. Statistical Analysis

The results of analysis of variance were expressed by mean ± S.D. SPSS 23.0 was used for statistical analysis by one-way analysis of variance with multiple comparison Tukey’s test. Dynamic variations in TVB-N, TPC and BA contents were processed using Microsoft Excel 2010 and Origin 9.1 Pro. Linear regression fitting was carried out using Origin 9.1 Pro. MetaboAnalyst v5.0 was adopted to construct a correlation heatmap for TVB-N, TPC, and BAs, while Cytoscape v.3.8.1 was used to draw the correlation network according to Pearson’s correlation coefficient.

## 3. Results and Discussion

### 3.1. Effects of Different Storage Temperature and Time on TVB-N and TPC of Yellowfin Tuna

**Figure 1:**
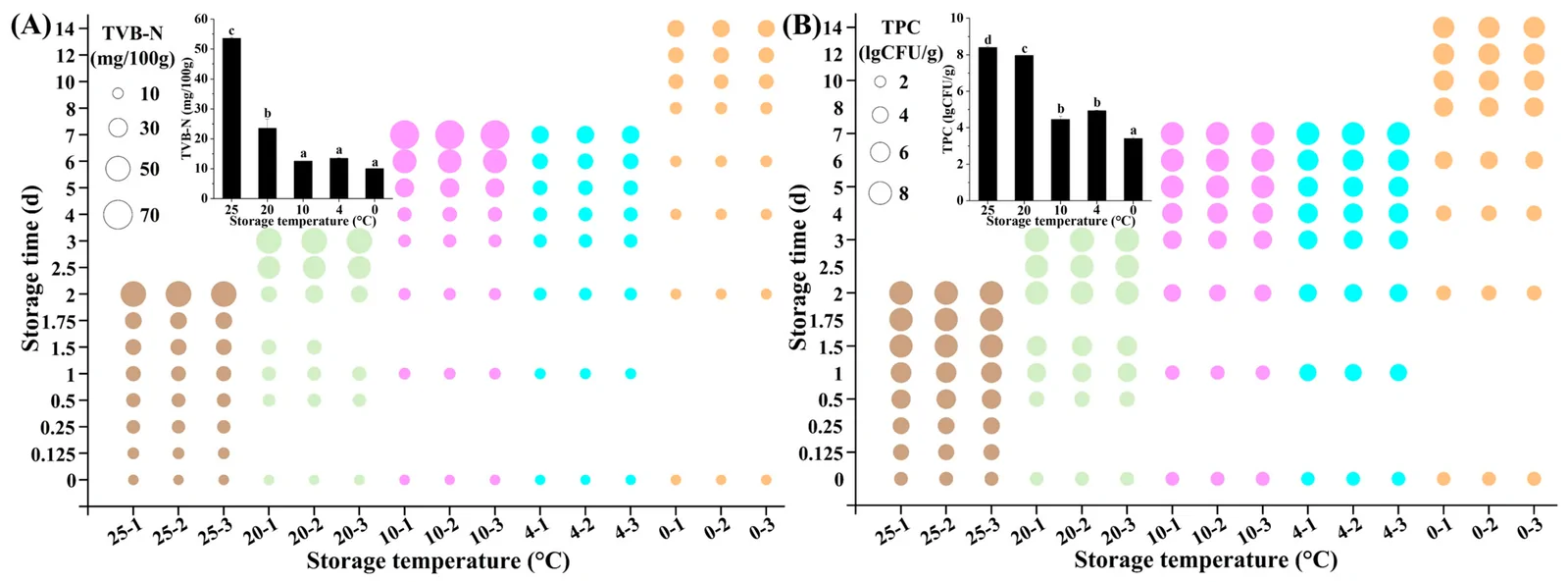
Effect of storage time on (**A**) TVB-N and (**B**) TPC in yellowfin tuna with different temperature treatments. The bar charts represent the quality changes in yellowfin tuna stored at different temperatures after 2 d of storage.The same applies hereafter. Different lowercase letters indicate significant differences among groups (*p* < 0.05).

TVB-N and TPC can serve as pivotal indicators to assess the freshness of yellowfin tuna [21]. The freshness of yellowfin tuna was excellent at the initial stage of storage under different temperature conditions. However, the freshness gradually declined with increasing levels of TVB-N and microorganisms during the storage process (Figure 1A). Overall, lower storage temperatures effectively retarded the accumulation of TVB-N and the proliferation of TPC. The standard limit of TVB-N content for tuna in China is 25 mg/100 g in the SC/T 3117-2006 [22]. In this study, the TVB-N value in yellowfin tuna stored at 25 °C, 20 °C, and 10 °C exceeded 25 mg/100 g after 2, 2.5, and 5 days, respectively. At the same time, the TVB-N value of yellowfin tuna stored at 0 °C and 4 °C did not exceed the limit during the experimental period, indicating that the lower temperatures delayed TVB-N accumulation and quality deterioration under the tested constant-temperature conditions. Moreover, the TVB-N value of yellowfin tuna decreased with decreasing storage temperature at the same storage time.

In this study, the changing trends of TPC and TVB-N were basically the same. The TPC values of tuna samples rose progressively with extended storage duration. According to the limit of 4 log CFU/g in the SC/T 3117-2006, the yellowfin tuna samples stored at 25 °C, 20 °C, 10 °C, 4 °C, and 0 °C exceeded the limit after 0.25, 1, 2, 1, and 6 days of storage, respectively. Similarly, TPC levels declined synchronously with the reduction in storage temperature under identical storage periods, and the TPC of yellowfin tuna stored at 10 °C, 4 °C, and 0 °C was significantly lower than those of samples stored at 25 °C and 20 °C. These results indicate that lower storage temperatures were associated with slower microbial proliferation and chemical spoilage under the present experimental conditions, and previous studies have also confirmed this point [9,23].

### 3.2. Effects of Different Storage Temperatures and Times on BAs of Yellowfin Tuna

**Figure 2:**
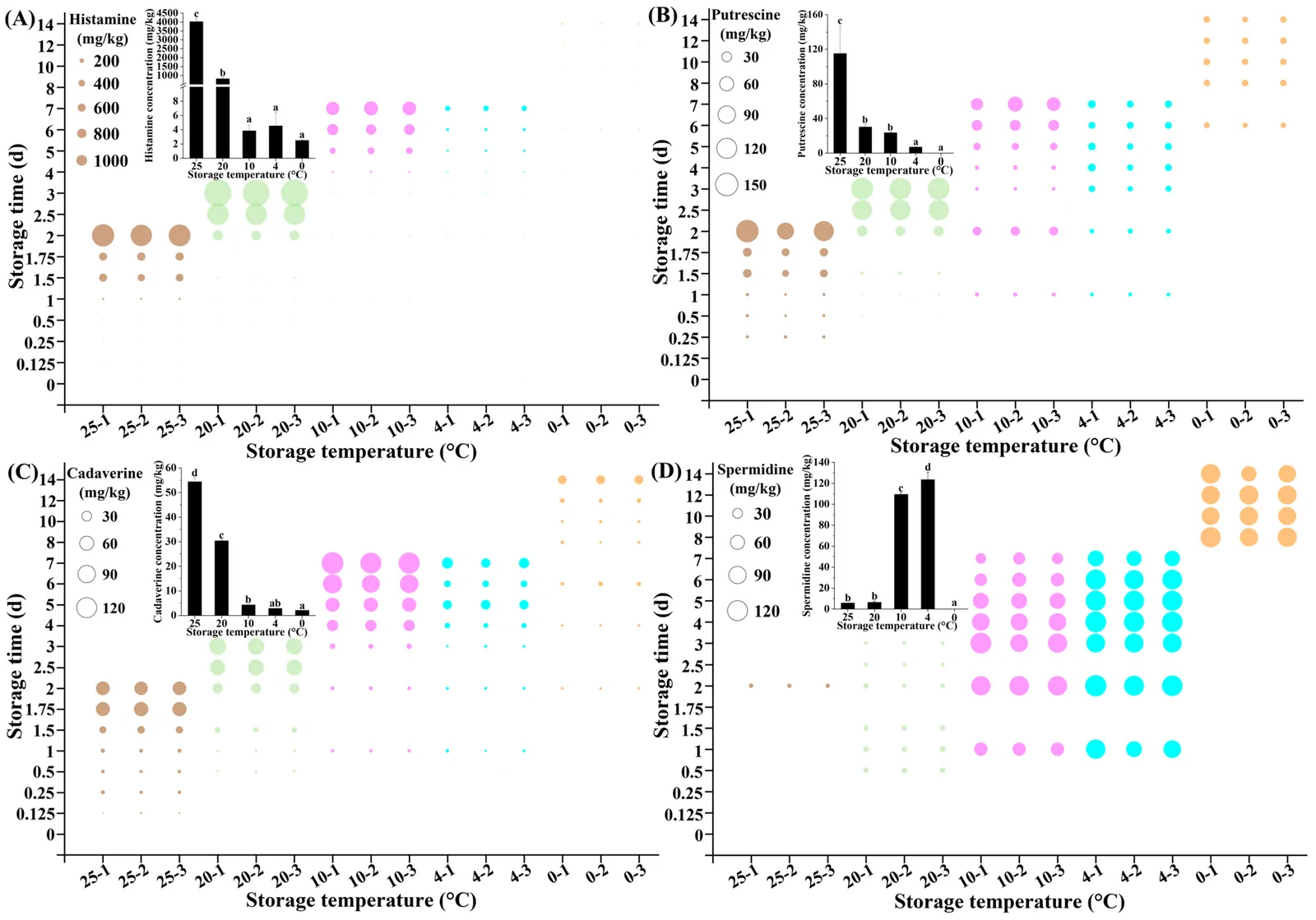
Effect of storage time on (**A**) histamine, (**B**) putrescine, (**C**) cadaverine, and (**D**) spermidine in yellowfin tuna with different temperature treatments. Different lowercase letters indicate significant differences among groups (*p* < 0.05).

**Figure 3:**
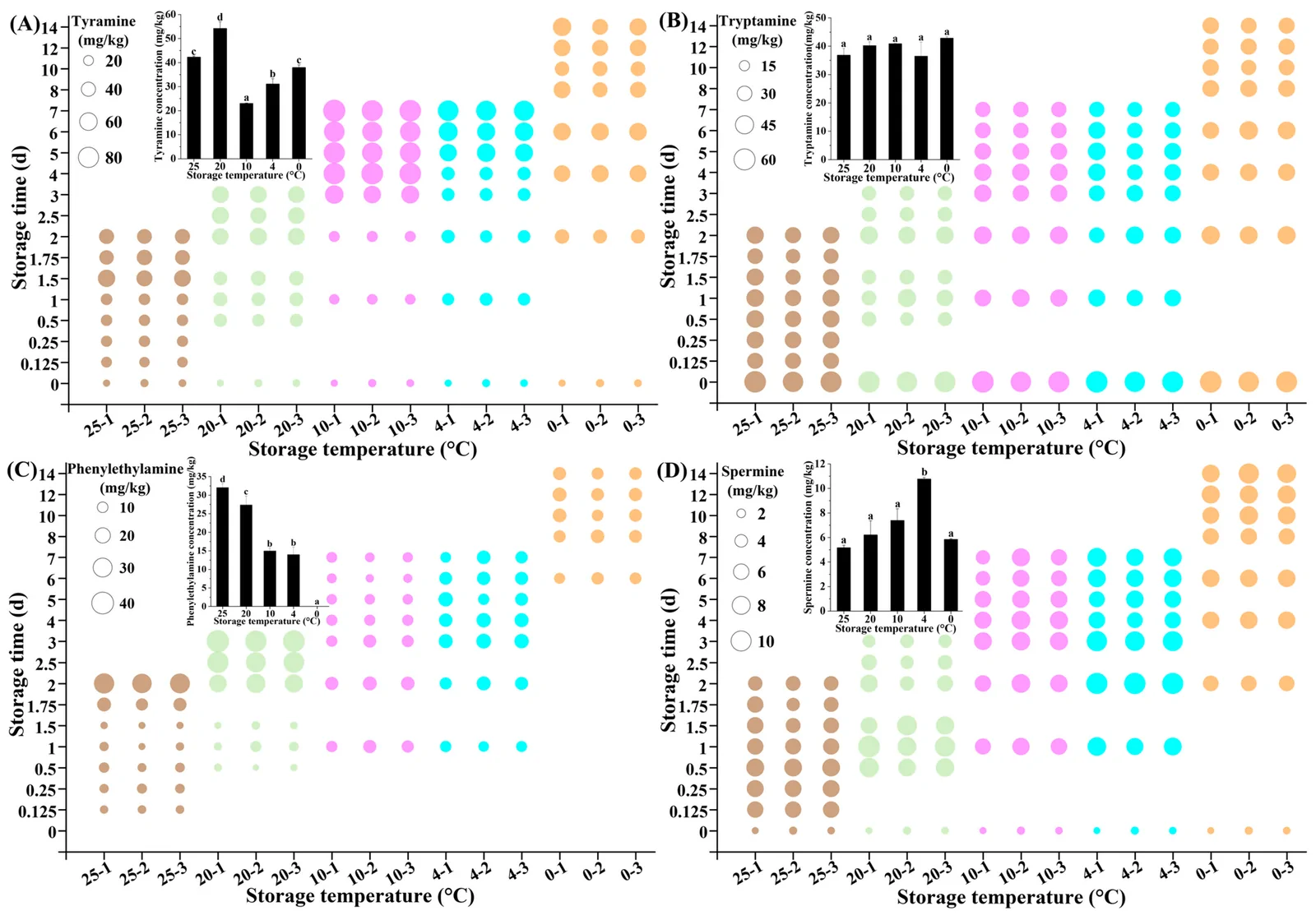
Effect of storage time on (**A**) tyramine, (**B**) tryptamine, (**C**) phenylethylamine, and (**D**) spermine in yellowfin tuna with different temperature treatments. Different lowercase letters indicate significant differences among groups (*p* < 0.05).

The harmful effects of BAs on human health cannot be ignored, and BA content in aquatic products is strictly limited all over the world, making the monitoring of BA content very important for the quality control of aquatic products [24]. In this study, histamine, putrescine, cadaverine, and spermidine were found at higher levels (Figure 2), and tyramine, tryptamine, phenylethylamine, and spermine were found at lower levels (Figure 3) during storage. The content of most BAs decreased as the storage temperature dropped, while spermidine gradually increased. The content of most BAs increased as the storage time lengthened.

Histamine was the most important BA in yellowfin tuna during storage. Histamine is usually formed by the conversion of histidine by histidine decarboxylase produced by histamine-producing bacteria, and the high content of histamine in seafood is one of the common causes of seafood poisoning in humans [8]. Overall, histamine content increased with storage time at different temperatures under the tested conditions. Specifically, at 25 °C storage, histamine content increased sharply after 1.5 days and reached 4019.1 mg/kg on day 2, greatly exceeding the limit of 90 mg/kg specified in the SC/T 3117-2006 (Figure 2A). Similarly, histamine levels in yellowfin tuna stored at 20 °C and 10 °C increased sharply on day 2 and day 5, respectively. Nevertheless, the histamine contents in yellowfin tuna stored at 4 °C and 0 °C were much lower than those at 25 °C, 20 °C and 10 °C, indicating that both 4 °C and 0 °C effectively reduce histamine content in yellowfin tuna, with 0 °C showing the strongest inhibitory effect on histamine accumulation. This result is closely related to the storage temperature of tuna and the growth of microorganisms. Most of the microorganisms capable of producing histamine are thermophilic bacteria. Low-temperature environments suppress the propagation of histamine-forming bacteria and reduce the activity of histidine decarboxylase, thereby inhibiting histamine biosynthesis [24].

Additionally, both cadaverine and putrescine are commonly associated with microbial activity, and they may enhance the toxicity of histamine at low concentrations [1,25]. In this study, the contents of putrescine and cadaverine increased with increasing storage time and were much lower than that of histamine in yellowfin tuna during storage (Figure 2B,C). Moreover, their contents generally decreased with decreasing storage temperature. Spermidine has been proposed as a potential spoilage marker because it forms at a faster rate than histamine during storage, as suggested in several articles [26,27]. However, at present, no threshold levels for these amines in food have been established [28]. Unlike the changing trends of the above BAs, spermidine was undetectable in tuna stored at 25 °C from day 0 to day 1.75 and was first detected at 5.77 mg/kg on day 2. (Figure 2D) The content of spermidine at 20 °C, 10 °C, and 4 °C first increased and then decreased with increasing storage time. At 0 °C, spermidine was detected after day 8, and there was also a slight downward trend in the later period. These results are partly consistent with previous research findings [27]. This may be attributed to the fact that the production of spermidine is related to the metabolism and cell growth of fish meat. Its concentration is higher in fresh products and decreases as the storage time increases. Moreover, its formation is not related to bacterial spoilage [29,30].

The changes in the four low-content BAs with temperature and time are displayed in Figure 3. The low-content BAs did not exhibit a consistent temperature-dependent trend at the same storage time. As shown in Figure 3A, the tyramine content in yellowfin tuna stored at 25 °C, 20 °C, and 10 °C increased with increasing storage time, while tyramine content at 4 °C and 0 °C decreased slightly on day 4 and day 10, respectively, and then continued to rise. On the second day, with the decrease in storage temperature, the tyramine content reached its maximum value at 20 °C. Interestingly, tryptamine content reached its peak of 57.56 mg/kg on day 0 during the entire storage process (Figure 3B). The tryptamine content slightly decreased during storage at different temperatures, and there was no clear change in tryptamine content with changes in temperature, indicating that tryptamine was less responsive to storage time and temperature under the present experimental conditions. Phenylethylamine was not detected on day 0, and its content increased with increasing storage time at 25 °C and 20 °C, while the content at 10 °C, 4 °C, and 0 °C first increased and then decreased (Figure 3C). Spermine existed at the lowest concentration among all quantified biogenic amines throughout the entire storage period (Figure 3D). Similar to spermidine, the content of spermine also first increased and then decreased slightly, and the cause of this phenomenon was the same as that for spermidine. With increasing storage time, spermine in yellowfin tuna reached a maximum of 10.78 mg/kg on day 2.

### 3.3. Correlation Analysis Among TPC, TVB-N, and BAs in Yellowfin Tuna at Different Storage Temperatures and Times

**Figure 4:**
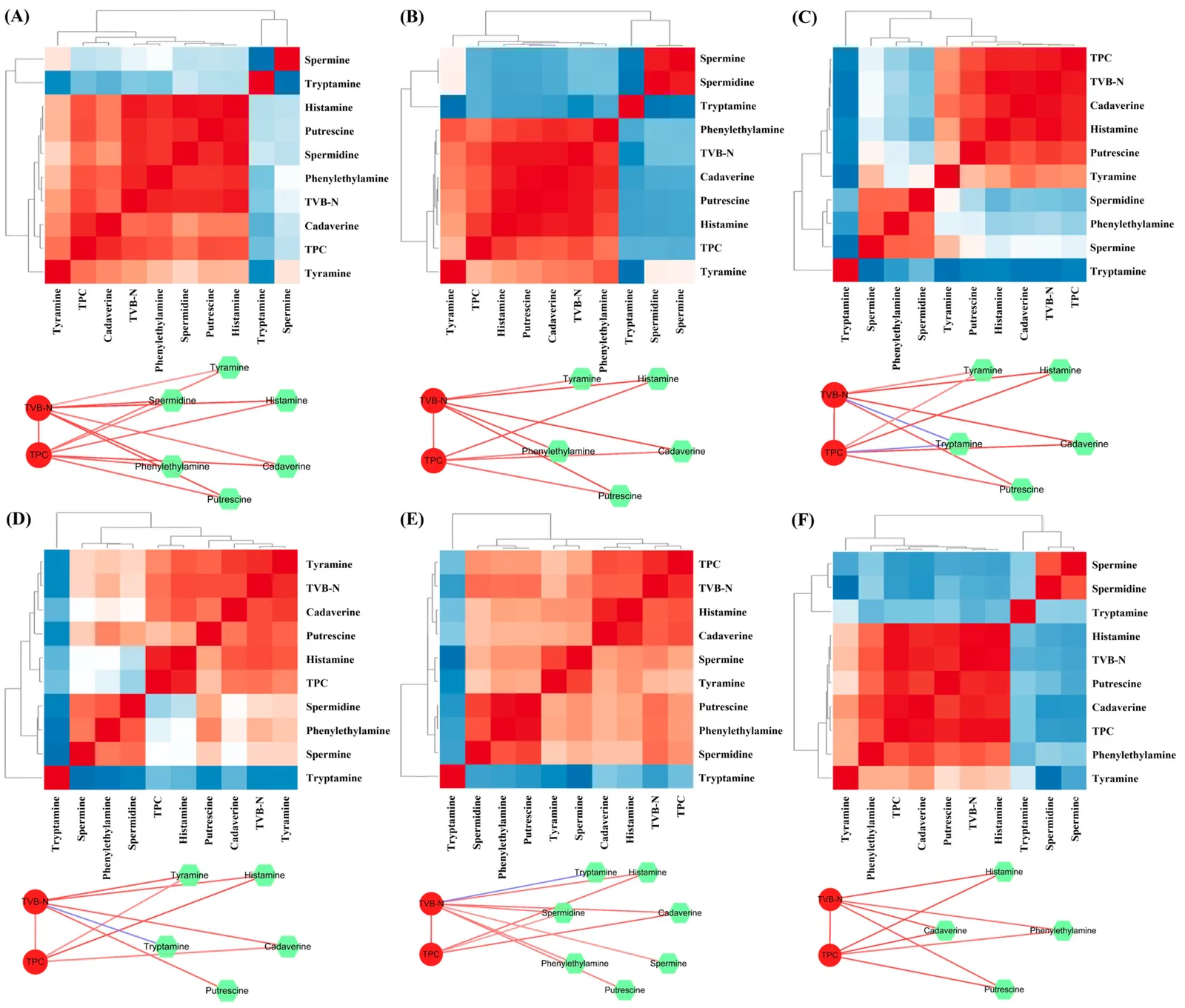
Correlation heatmaps and network diagrams of BA, TVB-N and TPC under different conditions: (**A**) 0 °C, (**B**) 4 °C, (**C**) 10 °C, (**D**) 20 °C, (**E**) 25 °C, and (**F**) day 2 in yellowfin tuna with different temperature treatments.

To identify candidate quality indicators for preliminary shelf-life estimation of yellowfin tuna, a correlation heatmap and network representing TPC, TVB-N, and BAs during storage were constructed (Figure 4). A correlation heatmap was generated using Pearson correlation coefficients, followed by hierarchical clustering. In the correlation heatmap, blue indicated a negative correlation between the two indicators, while red represented a positive correlation. Similarly, the blue and red lines in the correlation network diagram had the same meaning. The correlation networks were constructed according to the conditions of |r| ≥ 0.6 and *p* < 0.05.

The clustering patterns of the correlation heatmap in Figure 4 further reflected the temperature-dependent relationships among microbial growth, chemical spoilage, and biogenic amine accumulation. Because most quality indicators changed with storage time, some of the observed correlations may partly reflect shared temporal trends. At low storage temperatures, TPC, TVB-N, histamine, cadaverine, tyramine, phenylethylamine, and putrescine tended to cluster closely, indicating that their changes were relatively synchronized during storage within the present dataset. This grouping was relatively stable across the tested temperatures, suggesting that histamine and cadaverine were consistently related to the major spoilage indicators of yellowfin tuna. In contrast, some other biogenic amines, such as spermidine and spermine, showed less stable clustering patterns. As shown in Figure 4A–E, storage temperature significantly influenced the levels of nitrogenous compounds. At higher temperatures (20 °C and 25 °C), TVB-N and TPC exhibited strong positive correlations (r > 0.8) with most BAs, including histamine, putrescine, phenylethylamine, and cadaverine. These associations may be related to accelerated microbial growth and amino acid decarboxylation under elevated-temperature storage conditions [14]. However, as the storage temperature decreased to 10 °C, 4 °C, and 0 °C, the correlation pattern shifted. Notably, tryptamine and spermine displayed inconsistent or negative correlations with TVB-N and TPC (e.g., tryptamine at 10 °C, r = −0.626 to −0.673), suggesting that tryptamine and spermine were less suitable as candidate indicators for shelf-life estimation in this study because of their variable temperature-dependent changes [31]. On day 2, TPC, TVB-N, histamine, cadaverine, putrescine, phenylethylamine, and tyramine were grouped into a major positively correlated cluster, indicating that these variables co-varied with increasing storage temperature on day 2 (Figure 4F). For instance, histamine and cadaverine showed exceptionally high correlation coefficients (r > 0.78) across the entire temperature range from 0 to 25 °C. These correlations indicate the exploratory associations among different indicators, which are used to select appropriate shelf-life prediction indicators under experimental conditions.

In conclusion, although several BAs were generated during storage, histamine and cadaverine showed relatively stronger and more consistent associations with TPC and TVB-N than the other BAs under the tested conditions. Among them, histamine showed more pronounced changes in response to storage time and temperature than cadaverine. Therefore, histamine, TVB-N, and TPC were chosen for the shelf-life modeling and prediction in this study.

### 3.4. Establishment of Kinetic Model of Yellowfin Tuna During Storage

Kinetic modeling is a widely applied approach to characterize dynamic food quality changes during storage [32]. Considering statutory limit standards and spoilage sensitivity, histamine, TVB-N and TPC were selected as candidate indicators for preliminary kinetic modeling and shelf-life estimation. In the present study, the fitted equations were used to describe the quality changes observed within the same experimental batch. Kinetic equations are commonly used to predict the quality and shelf life of aquatic products, which follow zero-order or first-order reactions. In most studies, first-order kinetics are more widely applied to fit these changes under the tested conditions, expressed as:

(1) $$M=M_{0}e^{k_{s}t}$$

where *M*_0_ represents the initial quality parameter of yellowfin tuna, *M* represents the corresponding value after a storage duration of *t*, *k_s_* represents the reaction rate constant, and *t* represents the storage period (d).

Taking the logarithm on both sides of Equation (1) yields:

(2) $$\ln M=k_{s}t+\ln M_{0}$$

The Arrhenius equation is as follows:

(3) $$k_{s}=A_{0}exp(-\frac{E_{a}}{RT})$$

where *k_s_* represents the reaction rate constant; *A*_0_ represents the pre-exponential factor; *Eₐ* represents the activation energy (J/mol); *R* represents the universal gas constant (8.314 J/(mol·K)); and *T* represents the absolute temperature (K). The experimental temperatures of 0, 4, 10, 20, and 25 °C were converted to the corresponding absolute temperatures of 273.15, 277.15, 283.15, 293.15, and 298.15 K, respectively.

Taking the logarithm on both sides of Equation (3) yields:

(4) $$\ln k_{s}=\ln A_{0}-\frac{E_{a}}{R}\times \frac{1}{T}$$

According to Equation (4), a linear relationship is observed between *lnk_s_* and the reciprocal of the absolute temperature (1/*T*). After the apparent rate constants at different temperatures are determined, ln *k_s_* is plotted against 1/*T*, and a straight line with a slope of −*Eₐ*/*R* is obtained. From this linear relationship, the pre-exponential factor (*A*_0_) and activation energy (*Eₐ*) are calculated. Based on Equation (4), linear and nonlinear regression analyses were conducted respectively using Origin 9.0 on the quality index parameters such as histamine, TVB-N value, and TPC of yellowfin tuna to obtain the reaction rate constants *k_s_*, coefficients of determination *R*^2^, and regression equations of zero-order and first-order kinetic models, as shown in Table 1. The Σ*R*^2^ values can be used to compare the overall explanatory power of different kinetic models for changes in quality indicators at multiple storage temperatures. As can be seen from Table 1, most correlation coefficients *R*^2^ of the first-order kinetic model exceeded 0.9, suggesting that the first-order equations provided a good fit to the experimental data within the tested temperature range. The Σ*R*^2^ of the first-order kinetic coefficient was greater than that of the zero-order kinetic model, indicating that the first-order model more appropriately described the observed changes in quality indicators under the present experimental conditions. Therefore, the first-order kinetic model was selected for subsequent preliminary shelf-life estimation of yellowfin tuna.

Subsequently, by applying Equations (2) and (3), the predictive equations for the shelf life of yellowfin tuna were established.

(5) $$t_{L}=\frac{ln(\frac{M}{M0})}{A_{0}exp(-\frac{Ea}{RT})}$$

where *t_L_* represents the shelf-life equation of yellowfin tuna.

Subsequently, by applying Equation (3), the activation energies (*Eₐ*) for the variations in histamine, TVB-N, and TPC were calculated to be 79.50, 57.45, and 52.35 kJ·mol^−1^, respectively, while the pre-exponential factors (*A*_0_) were determined as 4.22 × 10^14^, 8.78 × 10^9^, and 7.60 × 10^8^. These parameters were then substituted into Equation (5) to derive Equations (6)–(8).

The model for predicting the shelf life based on histamine:

(6) $$t_{H}=\frac{ln(\frac{M}{M0})}{4.22\times 10^{14}exp(-\frac{79.50\times 10^{3}}{RT})}$$

The model for predicting the shelf life based on TVB-N:

(7) $$t_{T}=\frac{ln(\frac{M}{M0})}{8.78\times 10^{9}exp(-\frac{57.45\times 10^{3}}{RT})}$$

The model for predicting the shelf life based on TPC:

(8) $$t_{C}=\frac{ln(\frac{M}{M0})}{7.60\times 10^{8}exp(-\frac{52.35\times 10^{3}}{RT})}$$

In Equations (6)–(8), *t_H_*, *t_T_*, and *t_C_* represent the remaining shelf lives predicted by the models for histamine, TVB-N, and TPC of yellowfin tuna, respectively. *M* represents the measured value of histamine, TVB-N, or TPC after t days of storage, while *M*_0_ represents their corresponding initial measured values.

### 3.5. Prediction of the Shelf Life of Yellowfin Tuna

The estimated shelf-life endpoints based on histamine, TVB-N and TPC at different temperatures are shown in Table 2. According to the occupation standard SC-T 3117-2006 [22], the shelf-life endpoints of yellowfin tuna were defined as: Histamine = 90 mg/kg, TVB-N = 25 mg/100 g, and TPC = 4.0 log CFU/g. When any of these indicators exceeded its respective limits, the product was considered to have reached the end of its shelf life in the present study.

The shelf-life estimates derived from the TPC-based equation were generally shorter than those derived from the TVB-N- and histamine-based equations. This might be because the proliferation of microorganisms (TPC) usually occurs earlier than the large-scale accumulation of metabolic by-products, and TPC is more sensitive in reflecting the early spoilage of aquatic products [33]. The corresponding quality indicators were then measured at the calculated shelf-life endpoints, and the results are presented in Table S1. The relative deviations between the measured values of most indicators and their predefined limits were within 10%, suggesting that the predicted endpoints were generally consistent with the observed quality thresholds. Therefore, the proposed equations exhibited acceptable preliminary predictive performance under the tested constant-temperature conditions. In the present dataset, TPC reached the predefined limit earlier than TVB-N and histamine under most storage temperatures, suggesting that it was the earliest responsive endpoint indicator under the tested conditions. Therefore, TPC was used as a candidate indicator for preliminary shelf-life estimation in this study.

### 3.6. Investigation of the Relationship Between Quality Changes and the Shelf Life of Yellowfin Tuna

**Figure 5:**
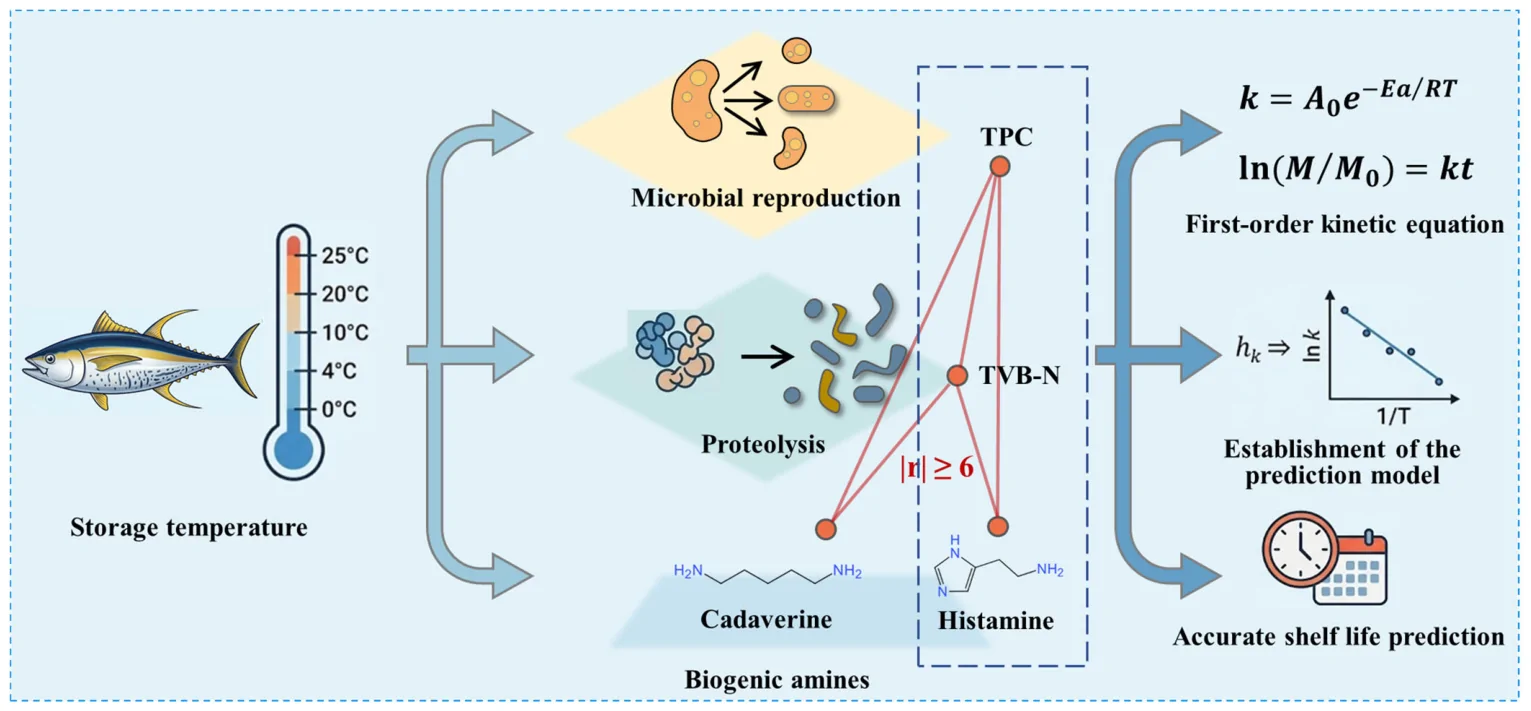
Changes in the quality of yellowfin tuna and establishment of the shelf-life model under different storage temperatures.

The changes in the quality of yellowfin tuna under different storage temperatures and the procedure used for preliminary kinetic assessment and shelf-life estimation are summarized in Figure 5. During the process of yellowfin tuna transportation, temperature fluctuations are inevitable. Especially during the process of thawing tuna for consumption, an increase in temperature is not conducive to maintaining the quality. Therefore, quality monitoring and shelf-life estimation are extremely necessary for yellowfin tuna at higher temperatures. In this study, the quality of yellowfin tuna stored at different temperatures deteriorated in all cases over time. This deterioration may be related to microbial growth, lipid oxidation, and protein degradation. Microbial proliferation may further promote protein hydrolysis and biogenic amine accumulation, thereby contributing to tuna spoilage [34,35].

Exploratory correlation analysis showed that TPC was associated with TVB-N, cadaverine, and histamine under the tested conditions. Therefore, these indicators had the potential to be used as indicators for predicting shelf life. Among the BAs, only the content of histamine changed significantly during storage. Based on the changes in TPC, TVB-N and histamine under different storage temperatures, first-order kinetic equations were developed to describe the observed quality changes and were used to estimate the shelf life at different temperatures. Because TPC was the first among the three indicators to reach its predefined critical limit during storage, the TPC-based equation provided the earliest estimated shelf-life endpoints under most tested conditions. Therefore, TPC was considered a candidate endpoint indicator for preliminary shelf-life estimation in this study. In this study, these equations provide preliminary kinetic estimates under the tested constant-temperature conditions.

## 4. Conclusions

This study demonstrated that storage temperature and duration markedly affected the microbial and chemical quality of yellowfin tuna. Total plate count (TPC), total volatile basic nitrogen (TVB-N), and most biogenic amines increased progressively during storage, with higher temperatures accelerating their accumulation. Histamine and cadaverine were strongly correlated with both TPC and TVB-N, indicating that these compounds can serve as sensitive indicators of quality deterioration. Histamine was selected as a candidate indicator for preliminary shelf-life estimation because it showed more pronounced changes during storage than the other biogenic amines. Preliminary kinetic equations based on histamine, TPC, and TVB-N were developed using first-order kinetics combined with the Arrhenius equation to estimate the shelf-life endpoints of yellowfin tuna under the tested constant-temperature conditions. The relative deviations between the measured values of most indicators and their preset limits were all within 10%, indicating that the models provided preliminary kinetic estimates. Among the tested indicators, TPC showed relatively greater temperature sensitivity within the present dataset. Overall, TPC showed the earliest response to quality deterioration within the present dataset and may serve as a candidate indicator for preliminary shelf-life estimation under the tested constant-temperature conditions. TVB-N, histamine, and cadaverine may provide complementary information for evaluating spoilage.

## Figures and Tables

**Table 1:** Reaction rate constant *K_s_* and determination coefficient *R*^2^ for zero- and first-order regression.

Index	Temperature/K	Zero-Order			First-Order		
		*k_s_*	*R* ^2^	Σ*R*^2^	*k_s_*	*R* ^2^	Σ*R*^2^
Histamine	273.15	0.7446	0.5840	3.0737	0.1425	0.7052	4.5288
	277.15	26.290	0.6049		0.7842	0.9659	
	283.15	202.12	0.6944		1.1147	0.9340	
	293.15	1920.2	0.7128		3.0945	0.9483	
	298.15	1222.2	0.4776		3.8826	0.9754	
TVB-N	273.15	1.0808	0.8888	4.1477	0.0724	0.9349	4.6045
	277.15	2.1826	0.9643		0.1392	0.9789	
	283.15	7.6750	0.7911		0.2852	0.9255	
	293.15	14.146	0.8603		0.5643	0.9541	
	298.15	14.310	0.6432		0.6151	0.8111	
TPC	273.15	0.3147	0.9758	4.7950	0.0667	0.9605	4.5909
	277.15	0.5999	0.9431		0.1167	0.8784	
	283.15	0.8443	0.9575		0.1625	0.9363	
	293.15	2.1154	0.9659		0.3851	0.9291	
	298.15	2.6573	0.9527		0.4739	0.8866	

**Table 2:** Prediction of the shelf life of yellowfin tuna at different temperatures based on the limit standards of histamine, TVB-N, and TPC.

Index	Temperature (K)	Predicted Shelf Life (d)
Histamine	273.15	16.87
	277.15	10.18
	283.15	4.90
	293.15	1.55
	298.15	0.90
TVB-N	273.15	11.28
	277.15	7.83
	283.15	4.62
	293.15	2.01
	298.15	1.35
TPC	273.15	4.40
	277.15	3.15
	283.15	1.95
	293.15	0.91
	298.15	0.64

## Data Availability

The original contributions presented in this study are included in the article/Supplementary Materials. Further inquiries can be directed to the corresponding authors.

## Supplementary Materials

The following supporting information can be downloaded at: https://www.mdpi.com/article/10.3390/foods15162829/s1, Table S1 Predicted shelf life and verification values of histamine, TVB-N and TPC at different temperatures.
