# Supplementary material for: Preliminary Kinetic Assessment of Quality Deterioration and Shelf-Life Estimation in Yellowfin Tuna Under Different Constant Storage Temperatures
Source: Foods. 2026 Aug 14;15(16):2829. doi: 10.3390/foods15162829 (PMC13511817; doi:10.3390/foods15162829)
Supplement: Supplementary file 1 [file foods-15-02829-s001.zip › foods-4441725-supplementary.pdf]

Supplementary Data

Table S1. Predicted shelf life and verification values of histamine, TVB-N and TPC at different temperatures.

Index	Limit standard	Temperature (K)	Predicted shelf-life (d)	Verification value	Relative error (%)
Histamine	90 mg/kg	273.15	16.87	91.35	1.50
		277.15	10.18	98.64	9.60
		283.15	4.90	92.29	2.54
		293.15	1.55	90.68	0.76
		298.15	0.90	83.54	-7.18
TVB-N	25 mg/100g	273.15	11.28	21.79	-12.84
		277.15	7.83	26.63	6.52
		283.15	4.62	25.26	1.04
		293.15	2.01	25.52	2.08
		298.15	1.35	23.78	-4.88
TPC	4.0 log CFU/g	273.15	4.40	4.02	0.50
		277.15	3.15	4.39	9.75
		283.15	1.95	4.09	2.25
		293.15	0.91	4.27	6.75
		298.15	0.64	4.16	4.00
